# Positive surgical margin is associated with biochemical recurrence risk following radical prostatectomy: a meta-analysis from high-quality retrospective cohort studies

**DOI:** 10.1186/s12957-018-1433-3

**Published:** 2018-07-03

**Authors:** Lijin Zhang, Bin Wu, Zhenlei Zha, Hu Zhao, Yuefang Jiang, Jun Yuan

**Affiliations:** Departments of Urology, Affiliated Jiang-yin Hospital of the Southeast University Medical College, Jiang-yin, 214400 China

**Keywords:** Positive surgical margin, Prostate cancer, Radical prostatectomy, Biochemical recurrence, Meta-analysis

## Abstract

**Background and purpose:**

Although numerous studies have shown that positive surgical margin (PSM) is linked to biochemical recurrence (BCR) in prostate cancer (PCa), the research results have been inconsistent. This study aimed to explore the association between PSM and BCR in patients with PCa following radical prostatectomy (RP).

**Materials and methods:**

In accordance with the guidelines of the Preferred Reporting Items for Systematic Reviews and Meta-Analyses (PRISMA), PubMed, EMBASE and Wan Fang databases were searched for eligible studies from inception to November 2017. The Newcastle–Ottawa Scale was used to assess the risk of bias of the included studies. Meta-analysis was performed by using Stata 12.0. Combined hazard ratios (HRs) and their corresponding 95% confidence intervals (CIs) were calculated using random-effects or fixed-effects models.

**Results:**

Ultimately, 41 retrospective cohort studies of high quality that met the eligibility criteria, comprising 37,928 patients (94–3294 per study), were included in this meta-analysis. The results showed that PSM was associated with higher BCR risk in both univariate analysis (pooled HR = 1.56; 95% CI 1.46, 1.66; *p* < 0.001) and multivariate analysis (pooled HR = 1.35; 95% CI 1.27, 1.43; *p* < 0.001). Moreover, no potential publication bias was observed among the included studies in univariate analysis (p-Begg = 0.971) and multivariate analysis (p-Begg = 0.401).

**Conclusions:**

Our meta-analysis demonstrated that PSM is associated with a higher risk of BCR in PCa following RP and could serve as an independent prognostic factor in patients with PCa.

**Electronic supplementary material:**

The online version of this article (10.1186/s12957-018-1433-3) contains supplementary material, which is available to authorized users.

## Background

Prostate cancer (PCa) is the most diagnosed malignancy and the second leading cause of cancer-related deaths among men in Western countries [1]. Radical prostatectomy (RP) has been shown to have a cancer-specific survival benefit for men with clinically localised PCa [2]. Although many patients are disease-free after surgery, nearly 30% [3] of patients still continue to experience biochemical recurrence (BCR). Defined as a detectable prostate-specific antigen (PSA) level following RP in the absence of clinical progression, BCR is the most common pattern of disease relapse [4]. Patients with BCR have a considerably worse prognosis, often develop metastasis, and can die of the disease [3, 4]. Therefore, identifying prognostic predictors of BCR after RP to assist clinicians in predicting outcomes for decision making is required.

Numerous nomograms including pathological tumour stage [5], Gleason’s score [6], seminal vesicle invasion [7], and lymphatic invasion [8] have been developed to predict subsequent risk of BCR after RP. Unfortunately, because the collective prognostic value of these factors is unsatisfactory, better biomarkers are urgently needed. Positive surgical margin (PSM) is defined as the histological presence of cancer cells at the inked margin on the RP specimen [9]. Although PSM is frequently reported in radical prostatectomy series, their clinical relevance remains uncertain despite extensive investigation. A number of studies have demonstrated an association between PSM and BCR [5, 10, 11], while others have observed insignificant or even contrary correlations [12–14].

Previously, Yossepowitch [15] systematically reviewed related studies on PSM reporting survival of surgical treatment for patients with PCa. These studies suggested that PSM in PCa should be considered an adverse oncological outcome. Nevertheless, a meta-analysis was not performed because of low-quality evidence and potential risks of bias. A meta-analysis utilises statistical methods to contrast and combine results from multiple studies, increasing the statistical power and reproducibility compared with individual studies [16]. Hence, to obtain the most conclusive results, we conducted a meta-analysis with high-quality retrospective cohort studies to assess the prognostic value of PSM in BCR.

## Methods

### Literature search

This meta-analysis was conducted in accordance with the Preferred Reporting Items for Systematic Reviews and Meta-Analyses (PRISMA) guidelines. A comprehensive search of the literature in PubMed, EMBASE, and Wan Fang databases up to November 2017 was performed using a combined text and MeSH heading search strategy with the following terms: (“prostate cancer” or “prostate AND neoplasms”) and (“radical prostatectomy”) and (“positive surgical margin”) and (“biochemical recurrence” OR “biochemical failure”). In addition, reference lists in the recent reviews, meta-analysis, and included articles were manually searched to identify related articles. The language of the publications was limited to English and Chinese.

### Inclusion and exclusion criteria

We defined the inclusion and exclusion criteria for study selection at the initiation of the search. The following inclusion criteria were used: (1) included definitive diagnosis of PCa and PSM assessed by pathologists; (2) all patients underwent RP treatment; (3) BCR after RP was defined; (4) the risk of BCR was estimated as hazard ratios (HRs) with corresponding 95% confidence intervals (CIs) or the risk could be calculated from the reported data; and (5) published in English or Chinese. The following exclusion criteria were used: (1) letters, reviews, case reports, editorials, and author responses; (2) non-human studies; (3) studies that did not analyse the outcome after PSM and BCR; (4) studies with duplicated patient populations that had been reported in previous publications; or (5) articles contained elements that were inconsistent with the inclusion criteria.

### Data extraction and quality assessment

Two investigators (Zhenlei Zha and Hu Zhao) independently extracted the data from all eligible publications. Any differences among evaluators were resolved by discussion with a third investigator (BinWu). The following data were extracted from the included studies using a standardised data collection protocol (Table 1**,** Table 2): first author’s name, year of publication, country, recruitment period, sample size, patient’s age, preoperative PSA level, Gleason score, pathological stage, positive percentage of PSM and BCR, definition of BCR, follow-up time, and the HRs (95% CIs) of PSM in univariate or multivariate Cox analyses for BCR. The quality of the eligible studies was evaluated according to the Newcastle–Ottawa Scale (NOS), which include three domains (selection of the study population, comparability of the groups, ascertainment of the outcome). We identified articles of “high quality” as those with NOS scores of 6–9, whereas scores of 0–5 were considered to indicate poor quality.

**Table 1:** Primary characteristics of the included studies

Author	Year	Country	No. of patients	Recruitment period	Age (years)	p-PSA (ng/ml)	Follow-up (months)	Surgical approach
Wettstein et al. [35]	2017	Switzerland	371	2008–2015	Median (range) 63 (41–78)	Median (range) 6.79 (0.43–81.4)	Median (range) 28 (1–64)	NA
Xun et al. [6]	2017	China	172	2003–2014	Median (IQR) 68 (62–72)	Median (IQR) 16.1 (10.9–28.3)	Median (IQR) 46.4 (33.4–62.4)	NA
Meyer et al. [36]	2017	Germany	903	1992–2005	Median (IQR) 63 (59–66)	Median (IQR) 6.4 (4.6–9.0)	Median (IQR) 133 (97–157)	NA
Gandaglia et al. [37]	2017	Multi-centred	94	2011–2015	Median (IQR) 64.3 (57.1–68.9)	Median (IQR) 9.7 (5.1–17.5)	Median (IQR) 23.5 (18.7–27.3)	Robot-assisted RP
Shangguan et al. [33]	2016	China	172	2003–2014	Median (range) 68 (62–72)	Median (range) 16.1 (10.9–28.3)	Median (IQR) 46.4 (33.4–62.4)	Open and laparoscopic RP
Zhang et al. [34]	2016	China	168	2006–2011	Median (range) 69 (53–85)	Median (range) 13.31 (4.59–36.12)	Median (range) 68 (7–98)	Laparoscopic RP
Simon et al. [12]	2016	Multi-centres	411	2001–2013	Mean ± SD 61 ± 6.1	NA	Median 63	NA
Sevcenco et al. [38]	2016	Multi-centres	7205	2000–2011	Median (IQR) 61 (57–66)	Median (IQR) 6 (4–9)	Median (IQR) 27 (19–48)	NA
Pagano et al. [20]	2016	USA	180	1990–2011	Median (range) 63.7 (58.8–67.6)	Median (range) 9.1 (6.3–17.1)	Median (range) 26.7 (8.8–66)	NA
Moschini et al. [39]	2016	USA	1011	1987–2012	NA	Median 12.0	Median 211.2	NA
Mortezavi et al. [40]	2016	Switzerland	100	1999–2007	Mean ± SD 63.5 ± 6.5	Mean ± SD 9.6 ± 8.3	Median (range) 126 (60–176)	Laparoscopic RP
Mao et al. [41]	2016	China	106	2008–2009	Mean (range) 68.1 (48–83)	Mean (range) 25.1 (3.1–104.3)	Median (range) 69 (8–84)	Laparoscopic RP
Whalen et al. [29]	2015	USA	609	2005–2011	Mean ± SD 61.2 ± 7.3	Mean ± SD 6.8 ± 6.3	Median (range) 20.5 (1–80)	NA
Song et al. [42]	2015	Korea	2137	1988–2011	Median (IQR) 67 (63–71)	Median (IQR) 6.9 (4.7–11.2)	Mean (range) 39.4 (8–1834)	NA
Reeves et al. [43]	2015	Australia	1479	2005–2012	Median 62	NA	Median 14	NA
Hashimoto et al. [5]	2015	Japan	837	2006–2013	Median (range) 65 (39–78)	Median (range) 6.9 (3–47.4)	Median (range) 20.5 (1.3–91.3)	Robot-assisted RP
Alvin et al. [44]	2015	Singapore	725	2003–2013	Median (range) 62 (37–79)	Median (range) 7.9 (0.79–72.9)	Mean (range) 28.5 (6–116)	Robot-assisted RP
Touijer et al. [13]	2014	USA	369	1988–2010	Median (IQR) 62 (57–66)	Median (IQR) 8 (5–15)	Median 48	NA
Ritch et al. [45]	2014	USA	979	2003–2009	Median 62	NA	Median 47	Open and robot-assisted RP
Kang et al. [21]	2014	Korea	3034	2004–2011	Mean ± SD 65.9 ± 6.6	Mean ± SD 11.6 ± 12.2	Median 47	NA
Fairey et al. [14]	2014	USA	229	1987–2008	Median (range) 65 (41–83)	NA	Median (range) 174 (2.4–253.2)	NA
Turker et al. [46]	2013	Turkey	331	1993–2009	Mean ± SD 62.79 ± 6.4	Mean ± SD 11.1 ± 10.5	Mean ± SD 29.7 ± 33.2	NA
Sammon et al. [10]	2013	USA	794	1993–2010	Mean ± SD 63.4 ± 8.1	Mean ± SD 5.6 ± 3.6	Median (IQR) 26.4(12.2–54.6)	NA
Chen et al. [30]	2013	China	152	2004–2011	NA	NA	Median (range) 48 (12–87)	Laparoscopic RP
Sooriakumaran et al. [11]	2012	Sweden	944	2002–2006	Median (IQR) 62.2 (58.2–65.8)	Median (IQR) 6.4(4.8–9.0)	Median (IQR) 75.6(67.2–86.4)	Robot-assisted RP
Lu et al. [31]	2012	China	894	1993–1999	Median (IQR) 62 (57–66)	Median (IQR) 6.0 (4.5–8.6)	Median (IQR) 9.9 (6.1–11.3)	NA
Iremashvili et al. [47]	2012	USA	1444	2003–2010	Mean (range) 61.3 (56–66.3)	Mean (range) 5.7 (4.5–8.0)	Median (range) 43.2 (3–216)	Open and robot-assisted RP
Connolly et al. [48]	2012	Australia	160	1988–1997	Mean ± SD 63.1 ± 6.3	Median (IQR) 9.95 (6.0–21.4)	Median (IQR) 26.2 (5.5–37.3)	Robot-assisted RP
Busch et al. [49]	2012	Germany	1845	1999–2007	Mean ± SD 62.0 ± 5.9	Median (range) 26.3 (17.0–42.1)	Median (range) 56 (0–35)	Laparoscopic RP
Berge et al. [50]	2012	Norway	577	2002–2008	Mean (range) 61.5 (42–76)	Mean (range) 8.4 (0.3–31)	Median (range) 36 (3–72)	Laparoscopic RP
Lee et al. [51]	2011	Korea	1000	2003–2009	Median (range) 66 (37–82)	Median (range) 7.8 (0.1–261.8)	Mean 39.4	NA
Alenda et al. [23]	2011	France	1248	1998–2008	Mean (range) 63 (44–78)	Mean (range) 10.9 (0.9–134)	Median 23.4	NA
Fukuhara et al. [52]	2010	Japan	364	2000–2009	Median (range) 66 (52–78)	Median (range) 8.1 (1.7–77.7)	Median (range) 33 (10–109)	NA
Cho et al. [53]	2010	Korea	171	2005–2009	Mean (range) 64.4 (49–80)	NA	Mean (range) 23.3 (2–51)	NA
Alkhateeb et al. [26]	2010	Canada	1268	1992–2008	Mean ± SD 62.0 ± 6.6	Median (range) 6.2 (0.1–65.9)	Mean (range) 78.1 (3–192)	NA
Jeon et al. [54]	2009	Korea	237	1995–2004	Mean (range) 64.5 (44–86)	Mean (range) 11.5 (0.2–98)	Median (range) 21.6 (2–88)	NA
Schroeck et al. [55]	2008	USA	3194	1988–2007	Median (IQR) 62.6(57.2–67.9)	Median (IQR) 6.3(4.5–9.6)	Median 31.2	NA
Pavlovich et al. [56]	2008	USA	508	2001–2005	Mean ± SD 57.6 ± 6.7	Mean (range) 6.0 (0.3–27)	Median (range) 12 (2–52)	Laparoscopic RP
Hong et al. [57]	2008	Korea	372	2003–2007	Mean (range) 64.2 (37–72)	Mean (range) 8.7 (0.2–104.2)	NA	NA
Cheng et al. [8]	2005	Indiana	504	1990–1998	Mean (range) 62 (34–80)	NA	Mean (range) 44 (1.5–144)	NA
Shariat et al. [58]	2004	USA	630	1994–2002	Median (range) 60.9 (40–75)	Mean (range) 6.1 (0.1–99)	Median (range) 21.4 (1–101.3)	NA

*p-PSA* preoperative prostate-specific antigen, *SD* standard deviation, *IQR* interquartile range, *NA* data not applicable

**Table 2:** Tumour characteristics of the included studies

Author	Specimen GS ≦ 7/˃ 7	Staging system	T stage 1–2/3–4	SM+/ SM−	No. of BCR (%)	Definition of BCR
Wettstein et al. [35]	292 /79	WHO/ISUP 2016	263/108	133/238	49 (13.2%)	Rising and verified PSA levels > 0.1 ng/ml
Xun et al. [6]	131/41	TNM 2002	NA	62/110	80 (46.5%)	The date of the first PSA elevated to 0.2 ng/ml
Meyer et al. [36]	879/24	TNM 2002	903/0	37/206	137(15.2%)	PSA level of ≧ 0.2 ng/ml and rising after RP
Gandaglia et al. [37]	55/39	TNM 2002	22/72	30/64	24 (25.5%)	Two consecutive increases in PSA ≧ 0.2 ng/ml
Shangguan et al. [33]	131/41	NA	NA	62/110	NA	Two consecutive increases in PSA ≧ 0.2 ng/ml
Zhang et al. [34]	136/32	TNM 2012	NA	30/138	NA	First PSA elevated to 0.2 ng/ml
Simon et al. [12]	368/43	NA	NA	353/58	70 (17%)	Single PSA concentration of > 0.2, two concentrations at 0.2 ng/ml
Sevcenco et al. [38]	6645/560	TNM 2009	NA	6137/1074	798 (11.1%)	Two consecutive increases in PSA ≧ 0.2 ng/ml
Pagano et al. [20]	90/90	TNM 2002	NA	74/106	120 (66.5%)	Two postoperative PSA values of ≧ 0.2 ng/ml
Moschini et al. [39]	647/364	NA	355/657	566/445	697 (69%)	PSA 0.4 ng/ml or greater
Mortezavi et al. [40]	86/14	NA	79/21	25/75	12 (12%)	Two consecutive increases in PSA ≧ 0.2 ng/ml
Mao et al. [41]	78/28	TNM 2002	63/43	20/86	31 (29.2%)	Two consecutive increases in PSA ≧ 0.2 ng/ml
Whalen et al. [29]	516/93	TNM 1997	435/174	483/126	73 (12%)	Two consecutive increases in PSA ≧ 0.2 ng/ml
Song et al. [42]	1722/415	NA	1899/248	2132/13,433	466 (21.8%)	Greater than 0.2 ng/ml
Reeves et al. [43]	1306/142	NA	1042/454	390/1089	238 (20.5%)	Greater than 0.2 ng/ml
Hashimoto et al. [5]	634/373	WHO 2004	677/160	243/594	102 (12.2%)	Two consecutive increases in PSA ≧ 0.2 ng/ml
Alvin et al. [44]	663/58	TNM 2010	497/228	311/414	104 (14%)	Two consecutive increases in PSA ≧ 0.2 ng/ml
Touijer et al. [13]	184/185	TNM 2010	46/323	138/231	201 (54%)	PSA ≧ 0.1 ng/ml with confirmatory rise
Ritch et al. [45]	783/196	TNM 2002	955/24	335/644	317 (32.4%)	Greater than 0.2 ng/ml
Kang et al. [21]	2575/459	TNM 2009	NA	974/2060	NA	A serum PSA value of 0.4 ng/ml or greater after RP
Fairey et al. [14]	133/96	TNM 2002	0/229	105/124	83 (36.2%)	Detectable PSA (ng/ml) followed by two consecutive confirmatory (1988–1994: PSA ≧ 0.3; 1995–2005: PSA ≧ 0.05; 2006–present: PSA ≧ 0.03)
Turker et al. [46]	167/164	TNM 1994	NA	80/251	70 (21%)	Higher than 0.2 ng/ml on 2 separate measurements 1 month apart
Sammon et al. [10]	760/34	AJCC 2002	592/202	162/632	107 (13.5%)	Two consecutive increases in PSA ≧ 0.2 ng/ml
Chen et al. [30]	109/43	NA	0/152	27/125	80 (52.6%)	Two consecutive increases in PSA ≧ 0.2 ng/ml
Sooriakumaran et al. [11]	900/44	NA	651/230	194/704	135 (15.2%)	Greater than 0.2 ng/ml
Lu et al. [31]	796/98	TNM 2010	703/191	250/644	277 (31%)	PSA ≧ 0.1 ng/ml with confirmatory rise
Iremashvili et al. [47]	1286/258	NA	NA	479/965	210 (15%)	Greater than 0.2 ng/ml
Connolly et al. [48]	95/65	NA	65/95	60/100	88 (55%)	Greater than 0.2 ng/ml
Busch et al. [49]	1538/307	NA	1802/9	537/1308	450 (24.4%)	PSA ≧ 0.1 ng/ml with confirmatory rise
Berge et al. [50]	553/24	TNM 2002	441/136	168/409	91 (16%)	Two consecutive increases in PSA ≧ 0.2 ng/ml
Lee et al. [51]	236/764	NA	NA	337/663	99 (9.9%)	Two consecutive increases in PSA ≧ 0.2 ng/ml
Alenda et al. [23]	1248/0	NA	NA	400/843	176 (16.9%)	PSA > 0.2 ng/mL
Fukuhara et al. [52]	332/32	TNM 2002	275/89	157/207	66 (18.1%)	Two consecutive increases in PSA ≧ 0.2 ng/ml
Cho et al. [53]	153/14	TNM 2002	126/45	58/109	15 (8.8%)	A serum PSA value of 0.4 ng/ml or greater after RP
Alkhateeb et al. [26]	1159/109	NA	853/415	264/1004	NA	A serum PSA value of 0.4 ng/ml or greater after RP
Jeon et al. [54]	190/45	TNM 2002	145/92	86/151	67 (28.3%)	Two consecutive increases in PSA ≧ 0.2 ng/ml
Schroeck et al. [55]	2855/359	NA	1991/1166	982/2212	706 (25.7%)	Greater than 0.2 ng/ml
Pavlovich et al. [56]	494/14	TNM 2002	416/92	69/439	102 (20%)	Two consecutive increases in PSA ≧ 0.2 ng/ml
Hong et al. [57]	361/11	TNM 2002	371/0	46/326	NA	First value greater than 0.2 ng/ml
Cheng et al. [8]	410/94	TNM 1997	348/156	174/330	157 (21.2%)	Two consecutive increases in PSA ≧ 0.1 ng/ml
Shariat et al. [58]	565/65	TNM 1997	NA	179/451	80 (12.7%)	First value greater than 0.2 ng/ml

*GS* Gleason score, *SM+/SM* surgical margin positive/surgical margin negative, *BCR* biochemical recurrence, *NA* data not applicable

### Statistical analyses

All statistical analyses in this meta-analysis were performed by Stata 12.0 software (Stat Corp, College Station, TX, USA). The association between PSM and BCR outcome was presented as summary relative risk estimates (SRREs) and 95% CIs. Heterogeneity between studies was calculated by the chi-square-based *Q* test and *I*^*2*^. A value of *p* < 0.10 or *I*^*2*^ > 50% was considered as statistically significant heterogeneity. A random-effects model was used if heterogeneity was significant, and otherwise, a fixed-effects model was used. Sensitivity analysis was used to estimate the reliability of the pooled results via the sequential omission of each study. Subgroup analysis was performed to check whether the pooled HR was influenced by the region, publication year, mean age, sample size, mean preoperative PSA (p-PSA), median follow-up, and the cut-off value for BCR. To assess the stability of the combined HR, sensitivity analysis was performed by removing individual studies from the meta-analysis. Publication bias was assessed by funnel plots and was statistically determined by Egger’s linear regression. Statistical significance was defined as a two-tailed value of *p* < 0.05, except for the heterogeneity tests.

## Results

### Literature search and study characteristics

**Fig. 1:**
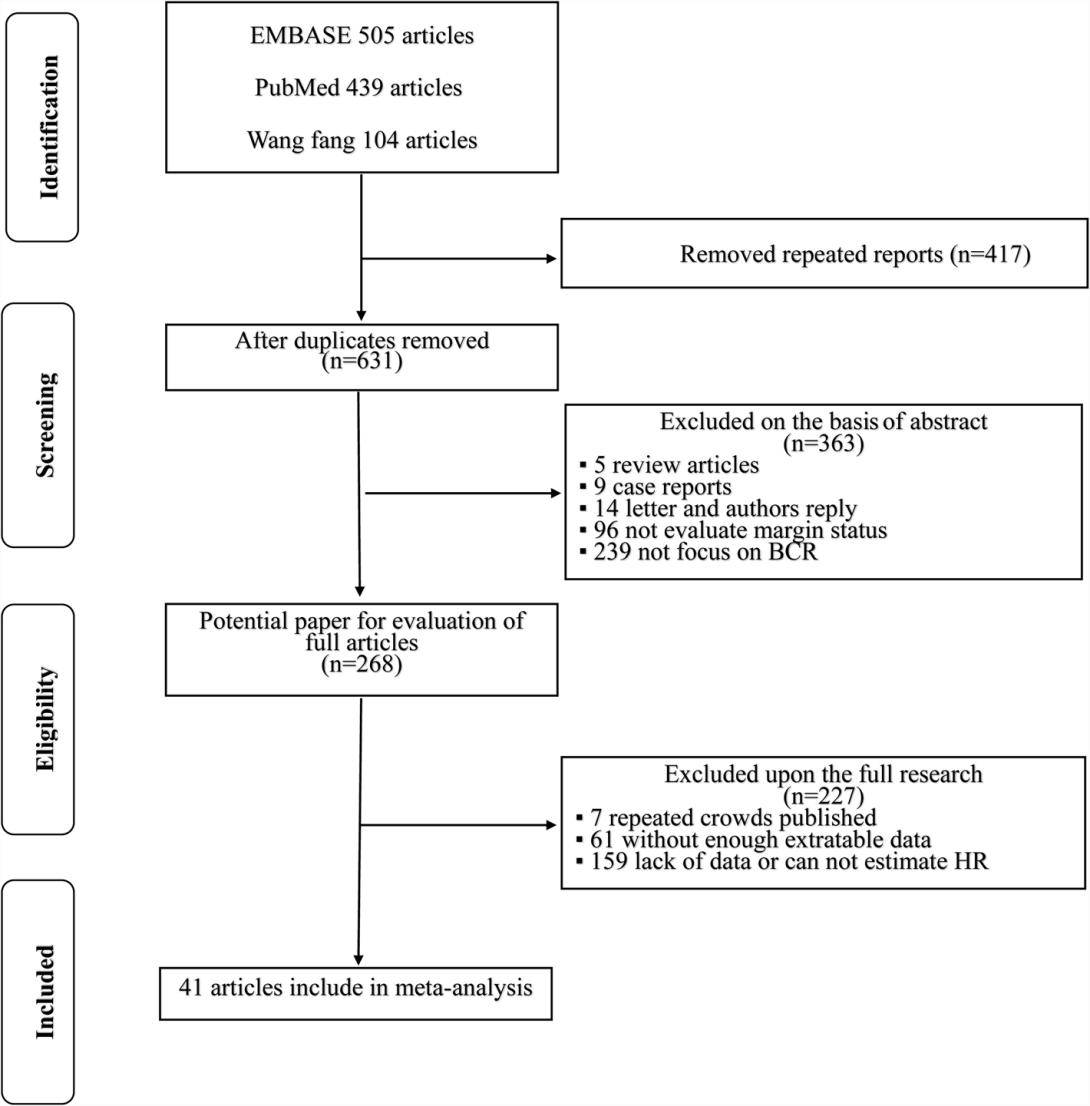
Flow diagram of the study selection process for this meta-analysis

The full process of the systematic literature review is shown in Fig. 1**.** In accordance with the PRISMA search strategy, 1048 relevant studies were initially identified. After carefully reading each article, 780 studies were excluded for the following reasons: duplicates, letters, or reviews; or contained no evaluated margin status and focus on BCR. After the remaining studies (*n* = 268) were reviewed, additional studies were excluded because certain cohorts were studied more than once or relevant data were lacking. Forty-one high-quality retrospective studies comprising 37,928 patients (94–3294 per study) were ultimately included in the meta-analysis.

The primary characteristics of the included studies are summarised in Table 1**.** All studies were published between 2004 and 2017. Of these, 19 studies were conducted in an Asian country, and 12 were conducted in North America; the rest were conducted in Europe (7) or in multiple countries (3). The median follow-up period of the studies ranged from 14 to 174 months. All included studies were published in English, except for two that were in Chinese. Of all of the studies, 8 used laparoscopic RP, 7 used robot-assisted RP, and 3 used open RP. BCR was defined using different cut-off values (0.1 ng/ml, 0.2 ng/ml, 0.4 ng/ml) among the included studies, and the incidence of BCR after RP ranged from 8.8 to 66.5% according to the reported values (Table 2). NOS [17] was applied to assess the quality of the included studies, and the results showed that all of the studies were of high quality with an NOS score ≥ 7. (Additional file 1: Table S1).

### Meta-analysis

**Fig. 2:**
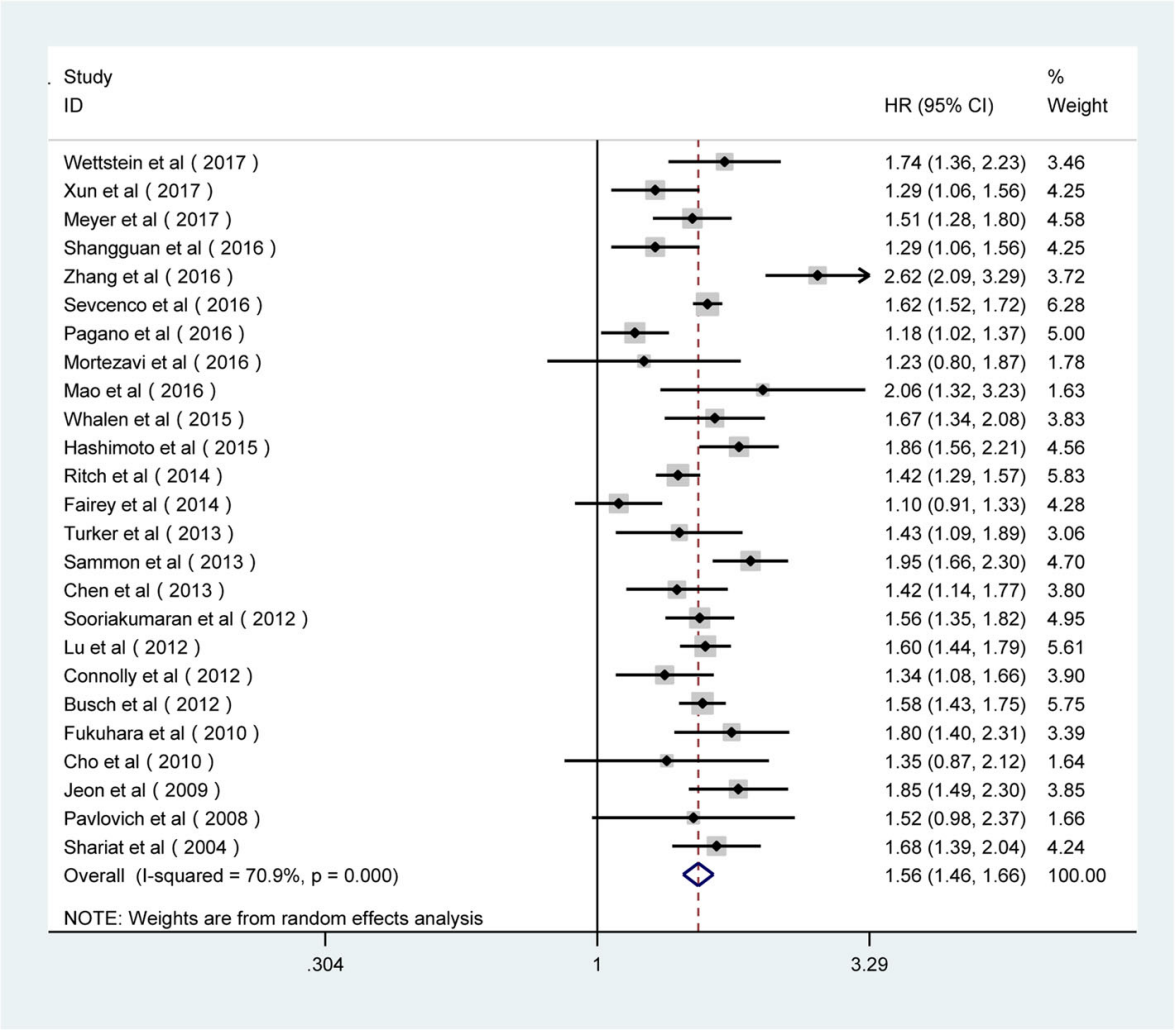
Forest plots of the association between PSM and BCR risk in the stratification analysis by univariate mode

**Fig. 3:**
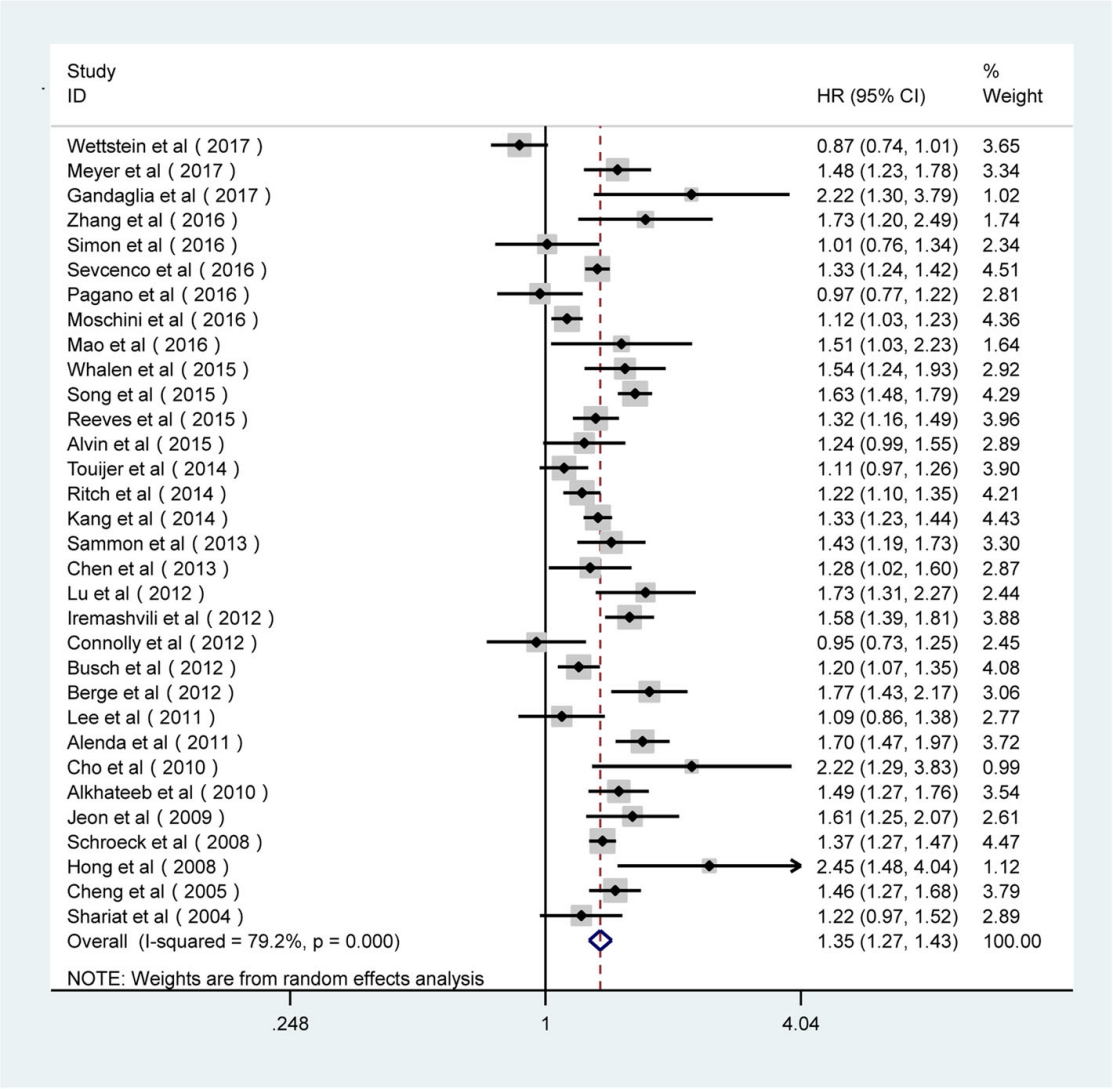
Forest plots of the association between PSM and BCR risk in the stratification analysis by multivariate mode

The forest plots of the meta-analysis in our study demonstrated that PSM was associated with poorer BCR in RP patients by univariate analysis (random-effects model, pooled HR = 1.56; 95% CI 1.46, 1.66; *p* < 0.001; Fig. 2) and multivariate analysis (random-effects model, pooled HR = 1.35; 95% CI 1.27, 1.43; *p* < 0.001; Fig. 3). Given the large heterogeneity between the studies, subgroup analyses were performed by region, publication year, mean age, sample size, mean preoperative PSA (p-PSA), median follow-up, and the cut-off value for BCR. Although no significant modifiers accounting for the inter-study heterogeneity were detected, the results of subgroup analyses were consistent with the primary findings (Table 3).

**Table 3:** Overall analyses and subgroup analyses for the included studies

Analysis specification	No. of studies	Study heterogeneity		Effects model	Pooled HR (95% CI)	*p* value
		*I*^2^ (%)	*p*_heterogeneity_			
Univariate analysis (BCR)						
Overall	25	70.9	< 0.001	Random	1.56 (1.46,1.66)	< 0.001
Geographical region						
Asia	12	72.1	< 0.001	Random	1.61 (1.43,182)	< 0.001
Europe and North America	12	70.8	< 0.001	Random	1.50 (1.37,1.65)	< 0.001
Date of publication						
≥ 2014	13	81.8	< 0.001	Random	1.52 (1.36,1.70)	< 0.001
< 2014	12	18.5	0.262	Fixed	1.61 (1.52,1.71)	< 0.001
Mean age (years)						
≥ 64	9	84	< 0.001	Random	1.62 (1.34,1.97)	< 0.001
< 64	15	55.6	0.005	Random	1.54 (1.45,1.64)	< 0.001
Sample size (cases)						
≥ 500	10	40.1	0.09	Random	1.61 (1.52,1.70)	< 0.001
< 500	15	76.9	< 0.001	Random	1.51 (1.33,1.71)	< 0.001
Mean p-PSA (ng/ml)						
≥ 10	7	81	< 0.001	Random	1.65 (1.38,1.97)	< 0.001
< 10	14	58.5	0.003	Random	1.59 (1.48,1.71)	< 0.001
Median follow-up						
≥ 36 months	11	77.1	< 0.001	Random	1.49 (1.33,1.67)	< 0.001
< 36 months	14	59.8	0.002	Random	1.61 (1.49,1.74)	< 0.001
BCR (ng/ml)						
Cutoff value 0.1	4	0	0.775	Fixed	1.61 (1.49,1.72)	< 0.001
Cutoff value 0.2	20	72	< 0.001	Random	1.58 (1.46,1.70)	< 0.001
Cutoff value 0.4	1	–	–	–	–	–
Multivariate analysis (BCR)						
Overall	32	79.2	< 0.001	Random	1.35 (1.27,1.43)	< 0.001
Geographical region						
Asia	14	67	< 0.001	Random	1.42 (1.29,1.55)	< 0.001
Europe and North America	15	84.7	< 0.001	Random	1.31 (1.19,1.43)	< 0.001
Multi-centred	3	71.9	0.029	Random	1.33 (1.00,1.78)	0.053
Date of publication						
≥ 2014	16	82.9	< 0.001	Random	1.27 (1.17,1.39)	< 0.001
< 2014	16	67.2	< 0.001	Random	1.44 (1.32,1.56)	< 0.001
Mean age (years)						
≥ 64	8	62.5	0.009	Random	1.56 (1.32,1.85)	< 0.001
< 64	22	81.5	< 0.001	Random	1.33 (1.24,1.43)	< 0.001
Sample size (cases)						
≥ 500	18	77.1	< 0.001	Random	1.40 (1.32,1.49)	< 0.001
< 500	14	76.8	< 0.001	Random	1.28 (1.12,1.47)	< 0.001
Mean p-PSA (ng/ml)						
≥ 10	7	80.8	< 0.001	Random	1.36 (1.22,1.57)	< 0.001
< 10	19	79	< 0.001	Random	1.35 (1.24,1.48)	< 0.001
Median follow-up						
≥ 36 months	16	79.6	< 0.001	Random	1.36 (1.24,1.46)	< 0.001
< 36 months	15	79.8	< 0.001	Random	1.34 (1.21,1.47)	< 0.001
BCR (ng/ml)						
Cutoff value 0.1	5	87.7	< 0.001	Random	1.22 (1.01,1.48)	0.044
Cutoff value 0.2	23	71.3	< 0.001	Random	1.39 (1.30,1.48)	< 0.001
Cutoff value 0.4	4	82.2	0.001	Random	1.34 (1.15,1.57)	< 0.001

### The sensitivity analysis and publication bias

**Fig. 4:**
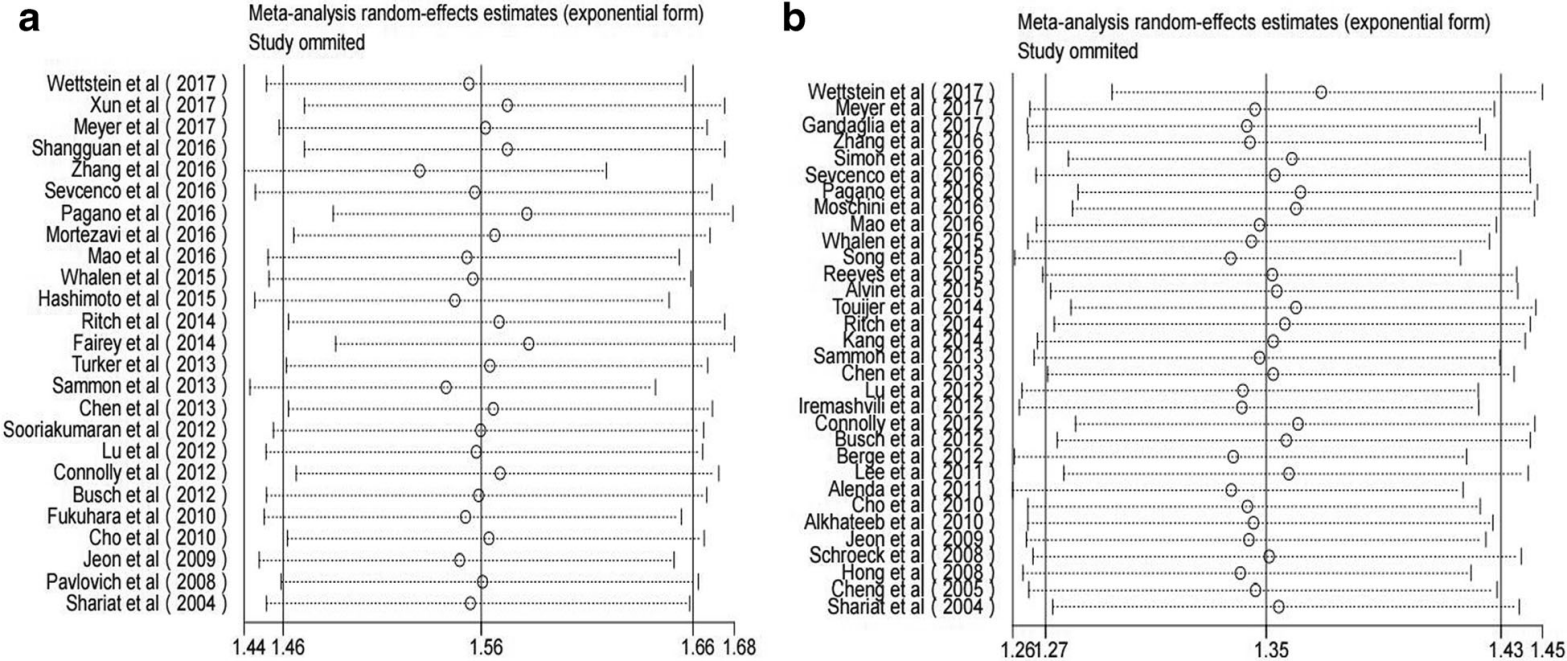
Sensitivity analysis of the association between PSM and BCR risk in PCa patients. **a** Univariate analysis mode. **b** Multivariate analysis mode

**Fig. 5:**
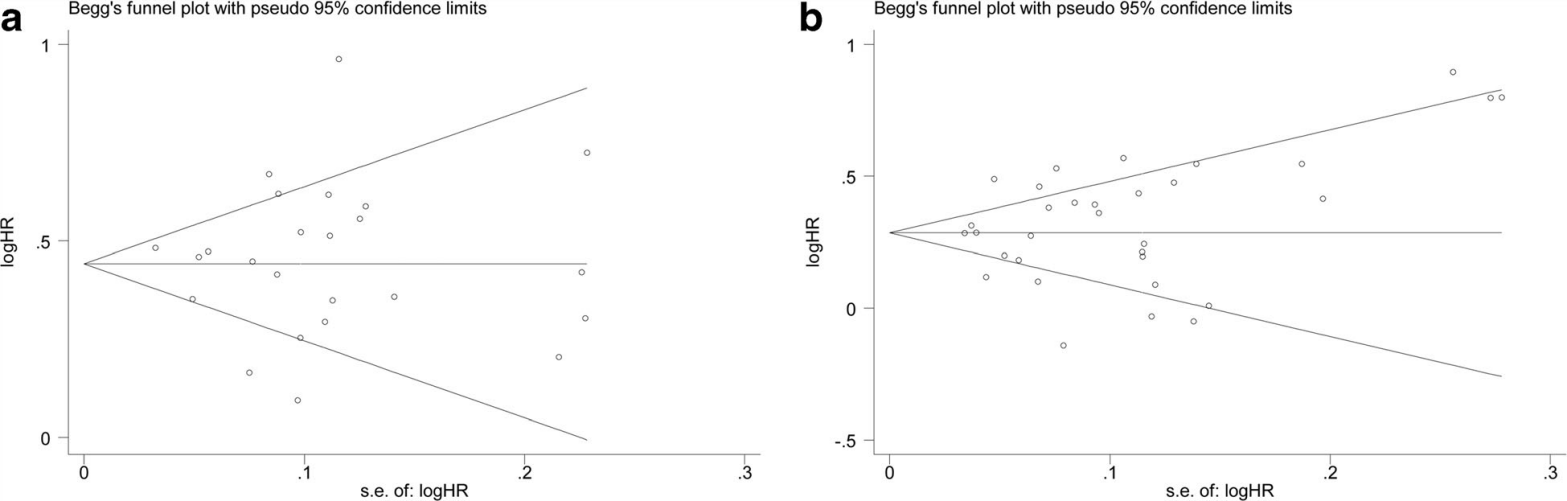
Funnel plots and Begg’s tests for the evaluation of potential publication bias. **a** Univariate analysis mode. **b** Multivariate analysis mode

With a sensitivity analysis, the overall significance did not change when any single study was omitted. The summary relative risk estimate (SRRE) for BCR ranged from 1.52 (95% CI, 1.44–1.62) to 1.58 (95% CI, 148–1.68) (Fig. 4a) in univariate analysis and 1.34 (95% CI, 1.26–1.42) to 1.37 (95% CI, 1.29–1.45) (Fig. 4b) in multivariate analysis. These results indicated that the findings were reliable and robust. To test for publication bias, Egger’s linear regression was performed. No significant publication bias was detected between these studies regarding HR of BCR in univariate analysis (p-Begg = 0.971; Fig. 5a) and multivariate analysis (p-Begg = 0.401; Fig. 5b), respectively.

## Discussion

With the increased public awareness and wide use of PSA-based screening, the number of patients diagnosed with PCa annually has been increasing [6]. Because RP provides superior cancer control and functional outcomes, this surgery has become a standard first-line treatment for eligible patients [18]. However, despite various advances in surgical technology, BCR has been reported in approximately 25–35% patients after RP and even more patients with intermediate–high risk [19]. Because BCR reportedly leads to distant metastasis and cancer death [20], it is necessary for men with BCR to undergo salvage radiation or hormonal therapy [11]. Therefore, identifying modifiable factors that affect the progression of BCR may help physicians in the selection of patients who are more likely to benefit from adjuvant multimodal therapy.

A number of nomograms have been developed to predict BCR after RP using either preoperative or postoperative variables [21]. Several clinical and pathologic factors have been included in these models, most of which cannot be altered by the treating physician (preoperative PSA [22], pathological T stage [5], pathological Gleason score [23]). The D’Amico risk stratification scheme [20] and Cancer of the Prostate Risk Assessment (CAPRA) score [24] have also been adopted in the urological community to predict the probability of BCR. Although these nomograms have been internationally validated, unfortunately, only a small number of them have predicted the probability of 5-year BCR with more than 70% accuracy [25]. Thus, efforts to improve existing outcome prediction tools for PCa are always encouraged.

PSM is a frequent situation encountered after radical prostatectomy (RP) for localised PCa with an occurrence ranging from 6 to 41% [9, 26, 27]. The incidence of PSM depends on various factors, including tumour biology, patient characteristics, pathological assessment method, and surgical technique [28]. We reported an overall PSM rate of 45.7% (17,339/37,928), which was slightly higher than other large series. Because the goal of surgical procedures is the complete removal of the tumour, the presence of PSM after RP is considered to be an adverse outcome associated with failure of the surgery to cure the PCa. However, the effects of PSM on clinical outcomes and the risk of BCR are still unclear. Several studies concluded that a PSM is an independent factor of BCR in patients with PCa after RP [11, 29–31]. However, not all patients with PSM show recurrence according to other studies [27, 28, 32]. Moreover, several reports showed that the effect of PSMs on prognosis depends on certain clinical and pathological features of the disease [26].

To the best of our knowledge, this study is the most up-to-date and informative meta-analysis on the association between PSM and BCR risk. The results obtained in our meta-analysis are in line with the previous systematic review by Yossepowitch et al. In addition, our study presented a series of advancements in comparison with previous studies. First, we included more eligible studies with high quality. The search by Yossepowitch et al. included studies up to 2013. However, our search included 21 additional studies published from 2014 to 2017, thereby improving the evaluation on the effect and enabling more subgroup analyses. In addition, the studies retrieved for our analysis were not limited to English; two Chinese articles [33, 34] also met the criteria for inclusion. Similar to Yossepowitch et al., we identified a significant relationship between PSM and BCR in RP. However, we also found that the pooled result of PSM had a large heterogeneity in both univariate (*I*^*2*^ = 70.9%) and multivariate (*I*^*2*^ = 79.2%) analyses. Even though the cut-offs varied among the included studies (0.1 ng/ml, 0.2 ng/ml, 0.4 ng/ml), the subgroup analyses achieved results similar to both univariate and multivariate analyses (Table 3). Meanwhile, the sensitivity analysis of our study revealed that the omission of each study did not have a significant impact on the merged value of HR.

However, several limitations of this study should be considered. First and foremost, all included studies were retrospective; therefore, the data extracted from those studies may have led to potential inherent bias. Second, the criteria to determine the presence of PSM in the pathological specimen were inconsistent in the included studies, which may have potentially contributed to heterogeneity. Thus, rigorous morphological criteria should be established to standardise the diagnosis of PSM. Third, substantial heterogeneity was observed in the meta-analysis, and although we used the random-effects model according to heterogeneity, it still existed in our studies. Moreover, from the subgroup analyses, we believed that the heterogeneity was caused by differences in factors such as patient and tumour characteristics. Finally, studies with negative results tend to be unsubmitted or unpublished; grey literature was not included, meaning that language bias may have been present in this study.

## Conclusions

In conclusion, this meta-analysis demonstrates that PSM has a detrimental effect on BCR risk in patients with PCa after RP and could therefore be considered to be an independent prognostic factor of BCR. Due to PSM’s excellent feasibility and low cost, this method should be more widely employed for BCR risk stratification and BCR prediction in patients with PCa. Given the inherent limitations of retrospective studies, further research is warranted, preferably with a longer follow-up period, to elucidate the potential role of PSM in influencing BCR risk.

## Additional file

Additional file 1: **Table S1.** Quality assessment of cohort studies included in this meta-analysis. (DOCX 20 kb)
