# Supplementary material for: Positive surgical margin is associated with biochemical recurrence risk following radical prostatectomy: a meta-analysis from high-quality retrospective cohort studies
Source: World J Surg Oncol. 2018 Jul 3;16:124. doi: 10.1186/s12957-018-1433-3 (PMC6029044; doi:10.1186/s12957-018-1433-3)
Supplement: Supplementary file 1 — Table S1. Quality assessment of cohort studies included in this meta-analysis. (DOCX 20 kb) [file 12957_2018_1433_MOESM1_ESM.docx]

Positive surgical margin is associated with biochemical recurrence risk following radical prostatectomy:

A meta-analysis from high-quality retrospective cohort studies

Lijin Zhang, Bin Wu, Zhenlei Zha^*^, Hu Zhao^*^, Yuefang Jiang^*^, Jun Yuan

**Table S1. Quality assessment of cohort studies included in this meta- analysis**

| **Study** | **Representativeness of the exposed cohort** | **Selection of the unexposed cohort** | **Ascertainment of exposure** | **Outcome of interest not present at start of study** | **Control for important factor or additional factor** | **Outcome assessment** | **Follow-up long enough for outcomes to occur** | **Adequacy of follow-up of cohort** | **Total quality scores** |
| --- | --- | --- | --- | --- | --- | --- | --- | --- | --- |
| Wettstein et al | ★ | ★ | ★ | ★ | ★★ | ★ | ★ | ★ | 9 |
| Xun et al | ★ | ★ | ★ | ★ | ★ | ★ | ★ | ★ | 8 |
| Meyer et al | ★ | ★ | ★ | ★ | ★★ | ★ | ★ | ★ | 9 |
| Gandaglia et al | ★ | ★ | ★ | ★ | ★ | ★ | ★ | ★ | 8 |
| Shangguan et al | ★ | ★ | ★ | ★ | ★ | ★ | ★ | — | 7 |
| Zhang et al | ★ | ★ | ★ | ★ | ★★ | ★ | ★ | — | 8 |
| Simon et al | ★ | ★ | ★ | ★ | ★★ | ★ | ★ | ★ | 9 |
| Sevcenco et al | ★ | ★ | ★ | ★ | ★★ | ★ | ★ | ★ | 9 |
| Pagano et al | ★ | ★ | ★ | ★ | ★★ | ★ | ★ | ★ | 9 |
| Moschini et al | ★ | ★ | ★ | ★ | ★ | ★ | ★ | ★ | 8 |
| Mortezavi et al | ★ | ★ | ★ | ★ | ★ | ★ | ★ | ★ | 8 |
| Mao et al | ★ | ★ | ★ | ★ | ★★ | ★ | ★ | ★ | 9 |
| Whalen et al | ★ | ★ | ★ | ★ | ★★ | ★ | ★ | ★ | 9 |
| Song et al | ★ | ★ | ★ | ★ | ★ | ★ | ★ | ★ | 8 |
| Reeves et al | ★ | ★ | ★ | ★ | ★ | ★ | — | ★ | 7 |
| Hashimoto et al | ★ | ★ | ★ | ★ | ★★ | ★ | ★ | ★ | 9 |
| Alvin et al | ★ | ★ | ★ | ★ | ★ | ★ | ★ | ★ | 8 |
| Touijer et al | ★ | ★ | ★ | ★ | ★ | ★ | ★ | ★ | 8 |
| Ritch et al | ★ | ★ | ★ | ★ | ★★ | ★ | ★ | ★ | 9 |
| Kang et al | ★ | ★ | ★ | ★ | ★ | ★ | ★ | — | 7 |
| Fairey et al | ★ | ★ | ★ | ★ | ★★ | ★ | ★ | ★ | 9 |
| Turker et al | ★ | ★ | ★ | ★ | ★ | ★ | ★ | ★ | 8 |
| Sammon et al | ★ | ★ | ★ | ★ | ★★ | ★ | ★ | ★ | 9 |
| Chen et al | ★ | ★ | ★ | ★ | ★★ | ★ | ★ | ★ | 9 |
| Sooriakumaran et al | ★ | ★ | ★ | ★ | ★★ | ★ | ★ | ★ | 9 |
| Lu et al | ★ | ★ | ★ | ★ | ★ | ★ | — | ★ | 7 |
| Iremashvili et al | ★ | ★ | ★ | ★ | ★ | ★ | ★ | ★ | 8 |
| Connolly et al | ★ | ★ | ★ | ★ | ★ | ★ | ★ | ★ | 8 |
| Busch et al | ★ | ★ | ★ | ★ | ★★ | ★ | ★ | ★ | 9 |
| Berge et al | ★ | ★ | ★ | ★ | ★ | ★ | ★ | ★ | 8 |
| Lee et al | ★ | ★ | ★ | ★ | ★ | ★ | ★ | ★ | 8 |
| Alenda et al | ★ | ★ | ★ | ★ | ★ | ★ | ★ | ★ | 8 |
| Fukuhara et al | ★ | ★ | ★ | ★ | ★ | ★ | ★ | ★ | 9 |
| Cho et al | ★ | ★ | ★ | ★ | ★★ | ★ | ★ | ★ | 9 |
| Alkhateeb et al | ★ | ★ | ★ | ★ | ★ | ★ | ★ | — | 7 |
| Jeon et al | ★ | ★ | ★ | ★ | ★★ | ★ | ★ | ★ | 9 |
| Schroeck et al | ★ | ★ | ★ | ★ | ★ | ★ | ★ | — | 7 |
| Pavlovich et al | ★ | ★ | ★ | ★ | ★★ | ★ | ★ | ★ | 9 |
| Hong et al | ★ | ★ | ★ | ★ | ★ | ★ | — | ★ | 7 |
| Cheng et al | ★ | ★ | ★ | ★ | ★ | ★ | ★ | ★ | 8 |
| Shariat et al | ★ | ★ | ★ | ★ | ★★ | ★ | ★ | ★ | 9 |
